# Non-tuberculosis extensive abdominal lymph node calcification leading to portal hypertension with esophageal and gastric variceal bleeding: a rare case report

**DOI:** 10.1186/s12876-022-02322-w

**Published:** 2022-05-15

**Authors:** Shuming Liu, Xingwu Xie, Xianzhi Tang, Huan He, Huiyuan Guan, Guangbin Chen

**Affiliations:** 1 Department of Medical Imaging Center, Renmin Hospital, Jinzhou Medical University Union Training Base, No. 39 Chaoyang Middle Road, Shiyan, 442000 Hubei China; 2 Department of Medical Imaging Center, Renmin Hospital, Hubei University of Medicine, No. 39 Chaoyang Middle Road, Shiyan, 442000 Hubei China; 3 Department of Hepatobiliary Surgery, Renmin Hospital, Jinzhou Medical University Union Training Base, No. 39 Chaoyang Middle Road, Shiyan, 442000 Hubei China

**Keywords:** Lymph node calcification, Portal hypertension, Esophageal and gastric variceal bleeding, Treatment method

## Abstract

**Background:**

Calcification of abdominal lymph node is a common clinical phenomenon, but it is extremely rare to cause serious adverse clinical outcomes. In the present case, the ruptured hemorrhage of the oesophagogastric fundic varices occurred as a result of portal hypertension due to compression of the portal vein by calcified lymph nodes. The patient was treated with medication, interventional therapy, endoscopic therapy, and surgery, respectively and the four different treatment options for the bleeding are worth summarizing. The etiology of this case is extremely rare and is the first to be reported in the world.

**Case presentation:**

A 32-year-old male patient with no apparent causes of sudden onset of vomiting of blood, the patient underwent four different treatment methods to stop the hemorrhage. The combined diagnosis of whole abdomen enhanced CT and angiography was calcified abdominal lymph nodes compressing the portal vein, leading to portal hypertension and resulting in esophageal and gastric variceal bleeding. Postoperatively, a biopsy of the caseous tubercular tissue of the abdominal wall observed intraoperatively was performed and the biopsy did not show a tubercular component. Therefore, the extensive intra-abdominal lymph node calcification was not associated with tuberculosis. The patient's bleeding ceased after surgery.

**Conclusion:**

This case has improved the clinician's understanding of the etiology of non-cirrhotic portal hypertension. Based on this, and with this case, the differences between various hemostatic measures were studied in depth.

## Background

Lymph node calcification is a common clinical phenomenon that occurs when calcium salts are deposited in the lymph nodes for various reasons. However, rupture of oesophagogastric fundic varices secondary to portal hypertension due to lymph node calcification compressing the portal vein is the first case in the world and is very rare. We recently treated a 32 year old patient with no previous history of any associated disease and report the following.

## Case presentation

A 32-year-old man presented to our hospital with a sudden onset of vomiting blood with no apparent cause. On admission, the vomiting of blood increased and was accompanied by black stools. The patient had no history of hepatitis, cardiac disease, tuberculosis or any surgical history. On physical examination, the patient had no fever, a heart rate of 86 beats per minute, a blood pressure of 130/80 mmHg and a respiratory rate of 20 breaths per minute. The patient was well developed and conscious with vague epigastric pain. All cardiopulmonary examinations were negative, there was no yellowing of the skin or sclera, no spider nevus, the liver and spleen were not palpable under the ribs, there was no percussion pain in liver and spleen, Murphy’s sign was negative, shifting dullness was negative, there was no edema in both lower limbs, bowel sounds were 5/min, there was no vascular murmur and all neurological investigations were normal.

**Fig. 1:**
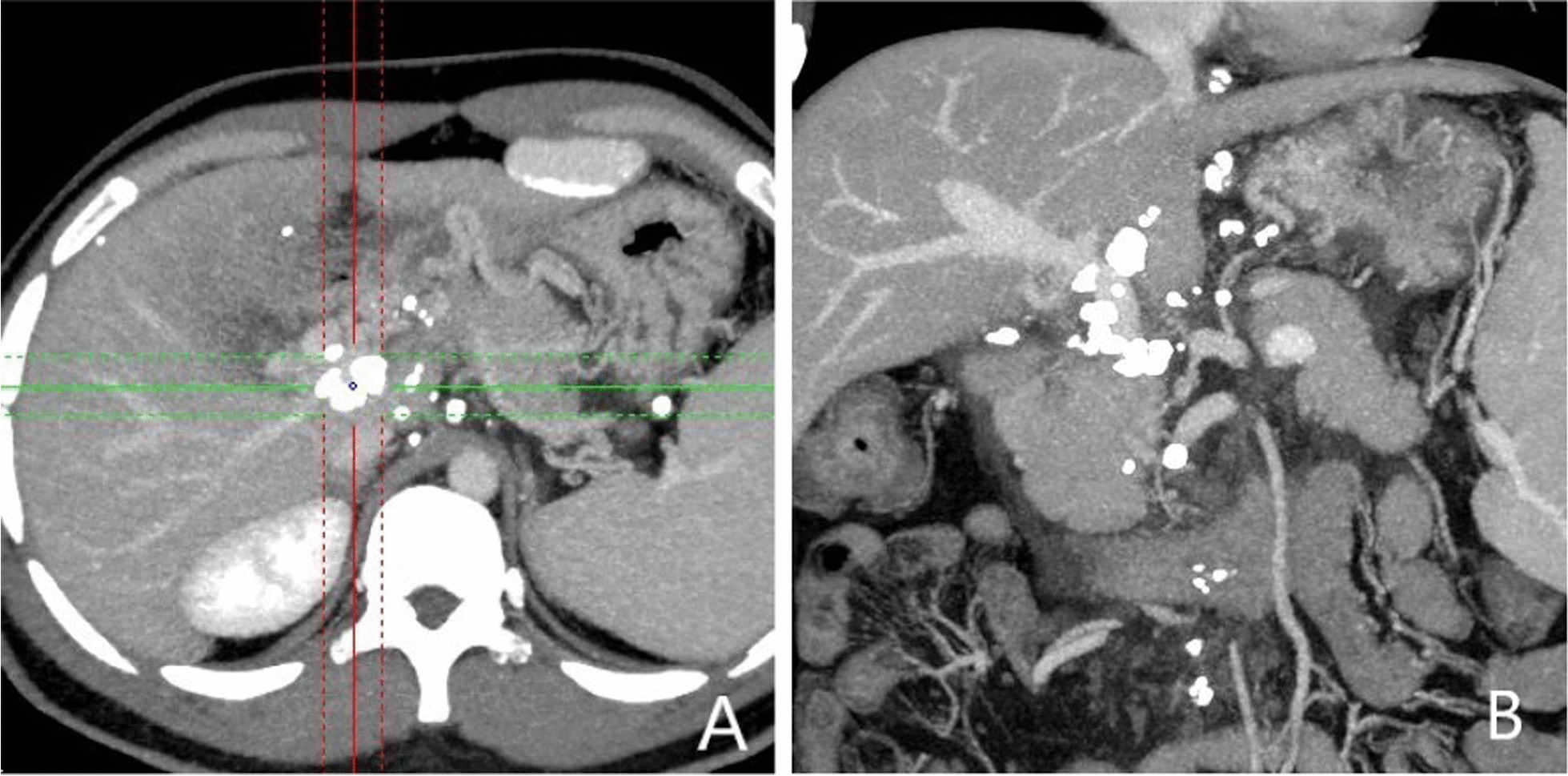
CT axial and coronal views: **A** and **B** Esophagogastric fundic varices with multiple lymph node calcifications in the abdominal cavity compressing the portal vein causing portal stenosis

**Fig. 2:**
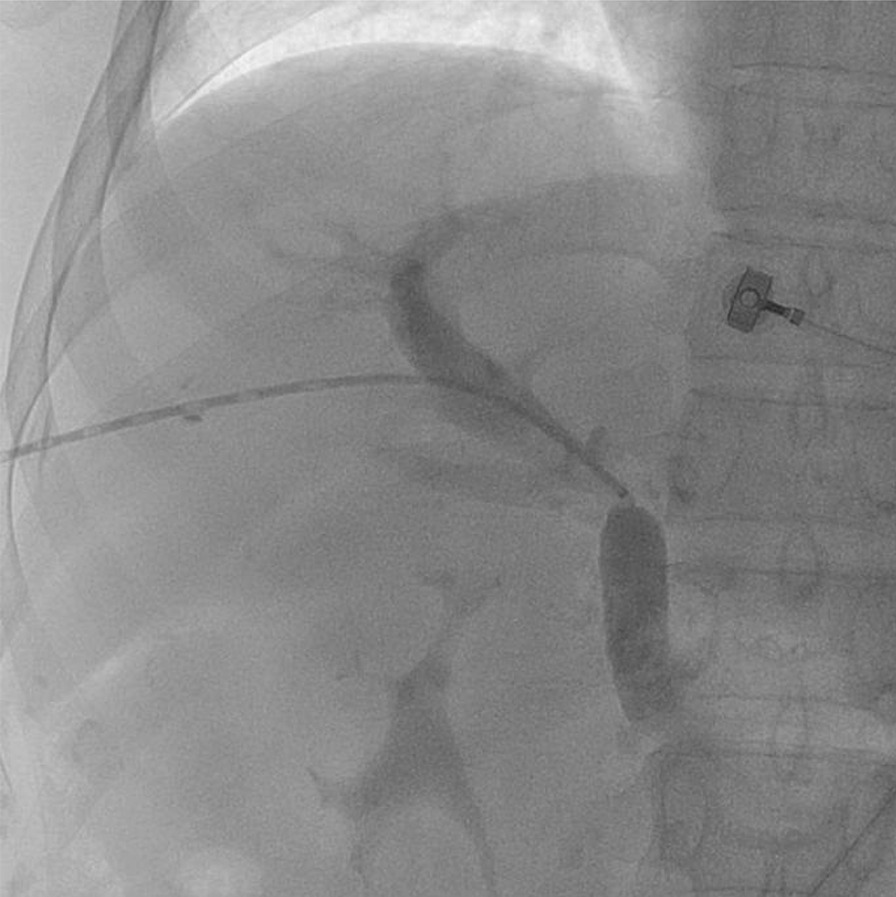
DSA shows severe stenosis of the right and left branches of the portal vein, consistent with the CT showing the site of compression of the lesion

**Fig. 3:**
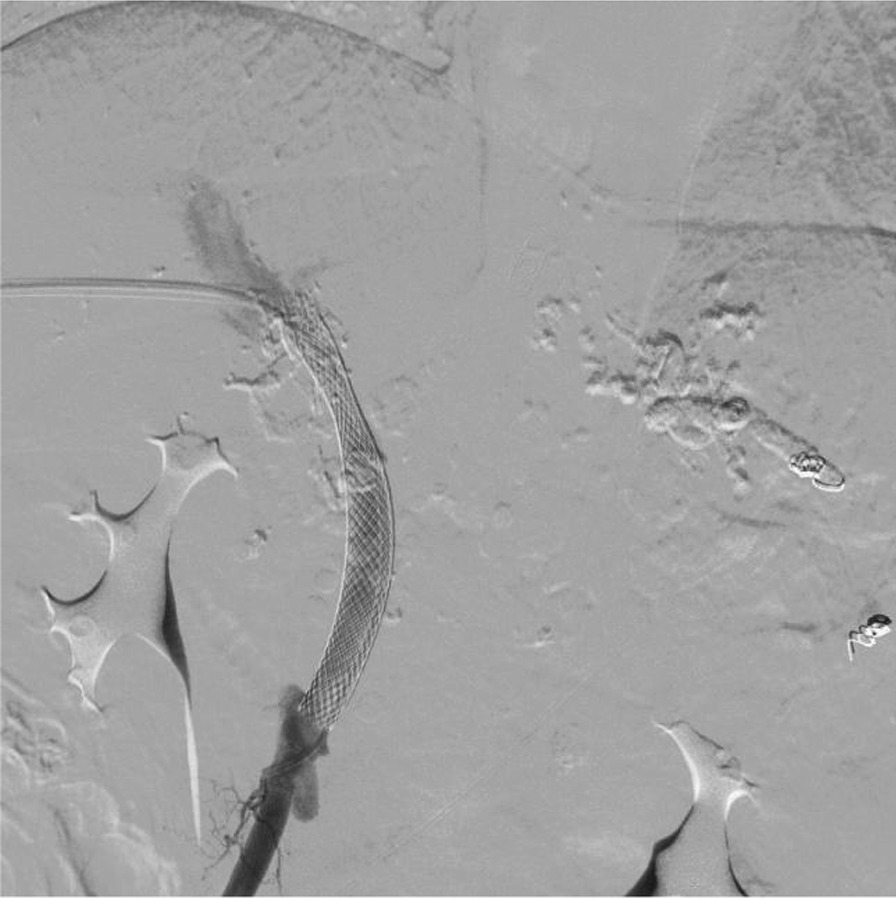
After portal vein stenting and PTVE embolization treatment

**Fig. 4:**
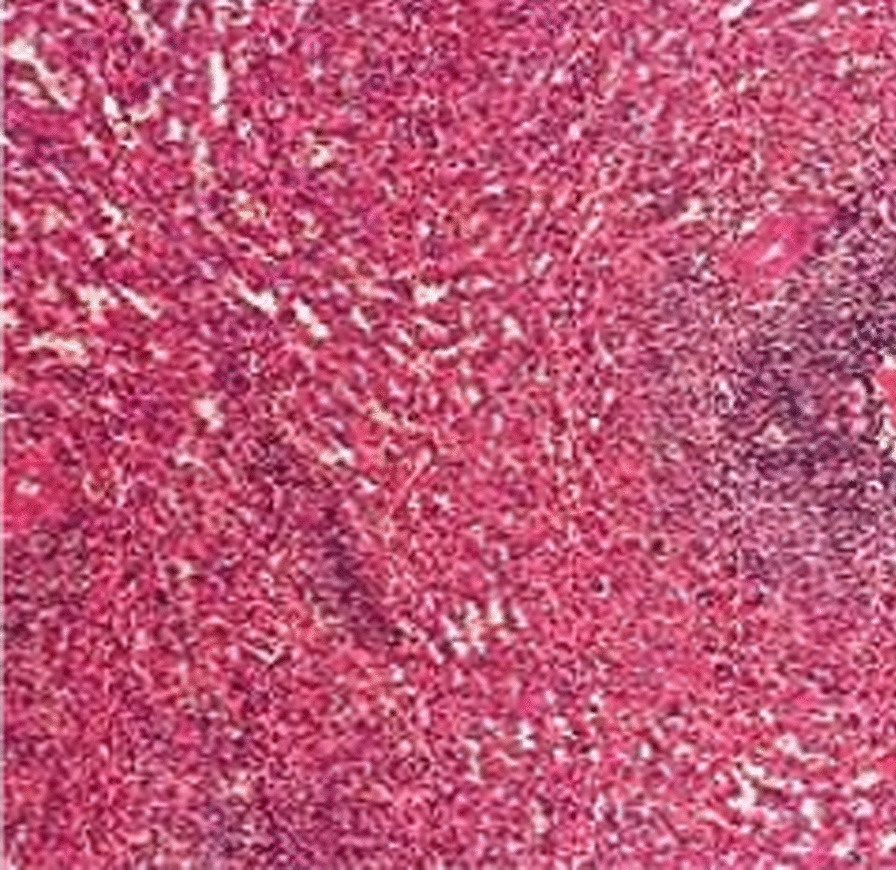
Caseous like mass in the abdominal wall with pathological findings (HE, × 400) and no tuberculosis component microscopically

**Fig. 5:**
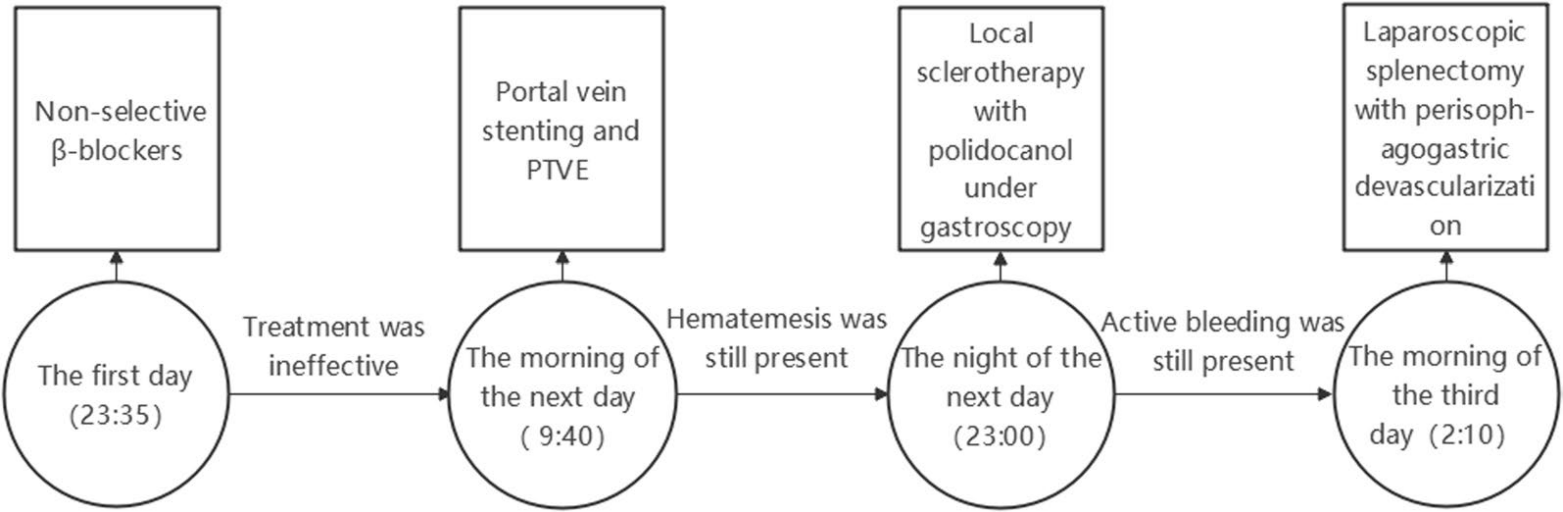
Time node flow chart of treatment measures implementation

Routine blood tests showed a significant decrease in hemoglobin concentration and red blood cell count. Laboratory investigations showed a prothrombin time of 14.1S, D-Dimer of 9.87 mg/L, hemoglobin of 86 g/L,blood urea nitrogen of 12.40 mmol/L, creatinine of 87.3 umol/L,

normal liver and renal function, normal electrolytes, negative hepatitis serology, normal autoimmune liver antibodies and negative for 9 tumor markers (male). ECG showed normal electrocardiographic changes. Ultrasonography showed splenomegaly. Enhanced CT of the whole abdomen showed varices of the lower oesophagogastric fundus; multiple intra-abdominal lymph nodes were calcified, with the larger lymph nodes located in the hepatic hilar region and compressing the main portal vein; and the spleen was enlarged (Fig. 1). No significant abnormalities were found on cranial and chest CT. Medication was given on admission to stop the bleeding, but there was no improvement. The morning of the next day, there was recurrent vomiting of blood, heavy bleeding and a significant drop in hemoglobin. Digital subtraction angiography (DSA) was then given and intraoperative arteriography showed no significant abnormalities; venography showed severe stenosis of the junction of left and right branches of the portal vein, with a stenosis of approximately 80%. A small amount of portal blood entered the liver through the stenotic portion and most of it flowed backwards into the splenic vein. Multiple side branches of the splenic and superior mesenteric veins formed and flowed into the gastro-Esophagus fundic plexus, and contrast spillage was seen in the fundic plexus (Fig. 2). Then, an appropriate amount of tissue glue and embolization coil was injected into the above-mentioned side branches for embolization, and portal vein stenting was performed at the portal vein stenosis (Fig. 3). The portal vein pressure below the stenotic segment was measured with a glass manometer at 34 cm H2O prior to embolisation. A combined whole abdomen enhanced CT and DSA angiogram showed lymph node calcification in the hepatic hilar region compressing the portal trunk, the same location as the DSA angiogram showed for the portal stenosis, so portal stenosis and portal hypertension were considered to be due to lymph node calcification compression. The night of the next day, the patient continued to vomit blood postoperatively. Emergency gastroscopy showed varices in the lower esophagus with multiple red signs; active bleeding was seen and was treated with local sclerotherapy with polidocanol under gastroscopy. However, there was still active hemorrhage after treatment, so laparoscopic splenectomy with perisoph-agogastric devascularization was performed in the early morning of the third day. Intraoperatively, there were multiple caseous nodules in the greater omentum and abdominal wall, and approximately 3 × 5 cm caseous nodules in the left abdominal wall. The greater omentum was clearly adherent to the abdominal wall and the liver and spleen, and the gastrointestinal tract was clearly dilated with dark red bloody material. There were no obvious cirrhotic changes in the liver, the spleen was markedly enlarged and the lower esophagus and perigastric fundus veins were markedly stagnant and dilated. The spleen and the caseous nodule in the left upper abdomen were excised and sent for pathological examination.Intraoperative diagnosis: EVB, hemorrhagic shock, abdominal tuberculosis and portal vein stenosis. Postoperative pathological examination of the spleen was consistent with chronic hematopoietic splenomegaly; the caseous mass of the abdominal wall showed a cystic wall-like structure microscopically, a large amount of necrotic material within the cyst, and vascular proliferation within the cystic wall with lymphocytic histiocytic infiltration (Fig. 4). Because of the intraoperative diagnosis of abdominal tuberculosis, postoperative culture of sputum acid-fast bacilli, ascites antacid cultures and tuberculosis DNA (sputum) tests were negative and combined with pathological examination suggested that calcification of the abdominal lymph nodes was not related to tuberculosis. Postoperatively, the patient's vital signs were stable, no significant drop in hemoglobin, no bleeding symptoms, bleeding stopped and he was discharged with symptomatic supportive treatment. Follow-up after discharge was good, with no complaints of bleeding (Fig. 5).

## Discussion and conclusions

The esophageal and gastric variceal bleeding (EVB) often result in a very high mortality rate due to the huge amount of bleeding and the extremely high recurrence rate [1]. The most common causes of EVB are prehepatic, hepatic and posthepatic, with cirrhosis being the most common of these, and non-cirrhosis-related diseases causing portal hypertension being extremely rare [2].

The diagnosis of the etiology of this case was difficult, as the patient was relatively young at 32 years of age. After various imaging studies, postoperative pathology and various laboratory tests, the possibility of portal hypertension due to cirrhosis, Buga syndrome, hepatic sinusoidal obstruction syndrome, thrombosis-related diseases, biliary system diseases, developmental variants, tumors and post-hepatic sources was gradually excluded. The etiology of this case was influenced by the presence of caseous tuberculosis-like tissue observed in the peritoneal cavity during surgery and by reports that abdominal tuberculosis can cause gastrointestinal hemorrhage in prehepatic portal hypertension, but subsequent laboratory tests related to tuberculosis and pathological biopsy directly excluded the case of abdominal lymph node calcification associated with tuberculosis [3]. Gastroscopy, multiple blood tests and a spleen biopsy also largely ruled out the possibility of a Dieulafoy lesion with hematological disease as the cause [4]. Therefore, in combination with the imaging data, the etiology of this case was diagnosed as prehepatic portal hypertension and EVB due to lymph node calcification compressing the portal vein, a very rare case the first of its kind in the world.As for what causes the formation of lymph node calcification, the author learned that the patients had intermittent mild abdominal pain and diarrhea symptoms for a long time before admission through telephone follow-up. In addition, CT examination in this case showed that there was a little effusion around pancreas and gallbladder fossa, blurred fat space at mesenteric root and multiple enlarged lymph nodes. Finally, combined with results from other researches, the author speculated that the etiology of lymph node calcification in this case may be chronic lymphadenitis leading to reactive hyperplasia of lymph nodes, and then with the self-healing mechanism of the body and the time goes on, calcium salt deposition appeared in lymph nodes [5].

Some studies suggest that a low hemoglobin level often exists when the value of blood urea nitrogen is at a high level. The high level of BUN comes from a large amount of hemoglobin produced during upper gastrointestinal bleeding. When this happens, it means that the patient's upper gastrointestinal bleeding is extremely serious, and it is necessary to immediately perform imaging examination or esophagogastroduodenoscopy to identify the bleeding site and immediately intervene to stop bleeding [6]. The BMN and Hb values in this case are in this situation.

In the case of EVB, treatment can be divided into: 1.Pharmacological treatment, commonly used drugs include somatostatin and non-selective β-blockers, among them, the mechanism of non-selective β-blockers is blocking β1 and β2 receptors to reduce portal vein pressure, so as to stop bleeding and prevent bleeding [7]. Some studies suggest that carvedilol, one of non-selective β-blockers, can block α1 receptor at the same time, thus significantly reducing portal vein blood perfusion and patient mortality [7]. However, this treatment is less effective in patients with larger amounts of bleeding, which was the case in this patient. 2. Endoscopic treatment: Endoscopic injection sclerotherapy (EIS), endoscopic variceal ligation (EVL) and endoscopic varicose vein tissue binder are commonly used. It is currently considered that emergency endoscopic haemostasis is the preferred treatment for EVB, which can achieve the elimination of varicose veins with a good prognosis, but if complete hemostasis is to be achieved, multiple and more complex operations are required, which can easily delayed resuscitation [8]. This case was treated with EIS after the intervention, but unfortunately it did not work. 3. Interventional treatment: There are currently two main procedures: transjugular intrahepatic portosystemic shunt (TIPS) and percutaneous transhepatic variceal embolization (PTVE). Studies have shown that TIPS is highly effective in stopping hemorrhage in cases of acute bleeding [9]. However, previous studies have concluded that TIPS can have a negative impact on the long-term prognosis of patients after the procedure and that TIPS is a difficult procedure to perform, making it difficult to generalise in the context of a large hepatitis population in China. PTVE is suitable for patients with aggressive bleeding due to its broad indications, and is relatively simple and less risky, and has a lower incidence of long-term hepatic encephalopathy than TIPS [10]. Due to the large amount of bleeding and severe stenosis of the portal vein, this patient was treated with a combination of portal vein stenting and PTVE. However, due to the large amount of bleeding and severe stenosis of the portal vein, multiple collateral branches of the splenic and superior mesenteric veins formed and merged into the fundic-esophageal vein to form multiple small veins, resulting in the embolic material failing to completely embolize the bleeding collateral branches of the fundic-esophageal plexus, leading to rebleeding after the intervention. 4. Surgery: Surgery is often not the first choice for EVB due to the high risk and relatively large trauma [11]^.^ In this case, the above three approaches failed to achieve good haemostasis and the patient was in critical condition, so laparoscopic splenectomy with perisoph-agogastric devascularization was performed. The patient did not bleed again postoperatively.

The etiology of non-cirrhotic portal hypertension is not fully understood, and this case is the first report of its kind in the world, raising our awareness of non-cirrhotic portal hypertension. In reviewing the diagnosis and treatment of this patient, we conclude that in EVB due to portal hypertension, the diagnosis of bleeding and screening for bleeding vessels is not difficult. Regardless of the etiology of the condition, the treatment method can be summarized as drug treatment, interventional, endoscopic, and surgical, with basically all the major hemostatic measures currently available being taken in this case, which is particularly rare and worth summarizing.

## Data Availability

Not applicable.

## Abbreviations

EVB: Esophageal and gastric variceal bleeding
TIPS: Transjugular intrahepatic portosystemic shunt
PTVE: Percutaneous transhepatic variceal embolization
EIS: Endoscopic injection sclerotherapy
EVL: Endoscopic variceal ligation
DSA: Digital subtraction angiography
